# Modulation of ASC-derived extracellular vesicles containing cargo that specifically enhances wound healing

**DOI:** 10.3389/fphar.2025.1635151

**Published:** 2025-09-15

**Authors:** Jodi P. Gurney, John W. Ludlow, Michael J. Van Kanegan, Robin L. Smith

**Affiliations:** ^1^ Exotropin, LLC, New York, NY, United States; ^2^ ZenBio, a BioIVT Company, Durham, NC, United States

**Keywords:** exosomes, regenerative medicine, wound healing, adipose-derived stromal/stem cells, diabetic ulcer, extracellular vesicles, therapeutic vehicle, mesenchymal stem cells

## Abstract

**Introduction:**

We have developed a bioreactor-based production system for manufacturing Human Adipose Stromal Cell (ASC) extracellular vesicles (EVs), which includes exosomes, using a highly controlled and tunable environment that can modify the cargo of these nanovesicles. The patented innovation focuses on engineering novel pro-healing EVs with therapeutic activity and using a topical formulation to treat diabetic ulcers.

**Methods:**

To evaluate biological activity of tuned ASC EVs, functional activity assays were performed using human primary dermal fibroblast and keratinocyte culture models. Molecular and biochemical assays were used to assess cytokine regulation, collagen production and cell migration. Rodent wound healing models were used to assess therapeutic potential of modified exosomes. A Human volunteer case study was carried out with a consenting individual suffering from chronic diabetic ulcers.

**Results:**

Herein we demonstrate that our proprietary engineered ASC EVs, eXo^3^ exosomes, contain a unique activity profile that reduces inflammatory cytokines, stimulates collagen production, as well as activates keratinocyte and fibroblast proliferation and migration. When formulated with an emollient and topically applied to an *in vivo* excisional wound model, tuned eXo^3^ exosomes demonstrated enhanced wound closure, increased keratinization, collagen deposition, and overall improved recover rate. In a clinical case study addressing non-healing diabetic foot ulcers, conducted under informed consent, topical treatment with tuned eXo^3^ exosomes formulated in a proprietary gel serum showed complete wound closure and dermal regeneration.

**Conclusion:**

Our current research efforts have developed an EV manufacturing system that can be directed to improve the healing capacity of ASC-derived EVs. We show enhanced wound healing and repair activity *in vitro* and *in vivo*. Our data supports the regenerative properties of exosomes and reinforces their strong therapeutic potential.

## 1 Introduction

Intercellular and extracellular communication is essential for maintaining homeostasis in multicellular organisms, especially in response to tissue injury. One key mode of communication involves extracellular vesicles (EVs) that serve as vehicles to facilitate the exchange of bioactive cargo, including proteins, RNAs, and lipids, that influence target cell behavior (Zhang et al., 2019). Current research has shown that EVs are secreted by all cells and provide paracrine communication by employing different targeting mechanisms as well as carry different cargos to effector cells (Gurunathan et al., 2021; Zou et al., 2023). The underlying processes involved in regulating secretion and packaging of vesicular content are not well understood, but harnessing these bioactive systems provides an attractive modality for enhancing the regenerative capacity of innate biological material. Many studies have demonstrated that EVs are differentially produced and secreted according to the cellular environment (Kowal et al., 2014; Pote and Gacche, 2025; Skotland et al., 2019). Our studies have built upon this premise by developing a bioreactor-based production system for manufacturing Human Adipose Stromal Cell (ASC) exosomes using a highly controlled and tunable environment that can modify EV production and cargo that reduces inflammatory cytokines, stimulates collagen production, as well as activates keratinocyte and fibroblast proliferation and migration (Zhang et al., 2015; Zhang et al., 2020). When formulated with an emollient and topically applied to an *in vivo* excisional wound model, tuned eXo^3^ exosomes demonstrated enhanced wound closure, increased keratinization, collagen deposition, and overall improved recover rate. In a clinical case study addressing non-healing diabetic foot ulcers, conducted under informed consent, topical treatment with engineered eXo^3^ exosomes formulated in a proprietary gel serum showed complete wound closure and dermal regeneration.

## 2 Materials and methods

### 2.1 ASC culturing and exosome purification

Research has shown that both exosome cargos and stem cell paracrine activity are dramatically altered by the environment, particularly with regard to the inflammatory and healing state of tissue (Shabbir et al., 2015; Shi et al., 2021; Rani and Ritter, 2016). Critical *ex vivo* environmental cues can tune stem cell-derived EVs to be pro-healing. Leveraging our expertise in stem cell biology, we have developed a proprietary bioreactor-based production system for manufacturing “tuned” EVs from adult stem cells. Modified EVs are termed “eXo^3^” throughout the manuscript and described in Section 2.9. By manipulating the cellular bioreactor environment, we have found that we can alter stem cell EV production and secretion as well as exosomal cargo packaging. Additionally, and most importantly, we have shown this manipulation of the bioreactor environment can improve the pro-healing capacity of the stem cell-derived EVs. Isolation and culturing of the Human Mesenchymal Stem Cells (MSC) subtype–adipose-derived stem cells (ASC), and exosome isolation and physical characterization, have been previously reported (Ludlow and Buehrer, 2024). The results within this report characterize enhanced features of eXo^3^ EVs by comparing to “native” EVs which were prepared in tandom from the same lot of ASCs cultured in an identical bioreactor system under traditional culturing conditions with no modification steps.

It is important to maintain a controlled environment while manufacturing EVs that retain wound healing properties. An aging microenvironment will promote cellular senescence that can shift EV production containing pro-inflammatory characteristics that negatively affect tissue repair and exacerbate cellular pathology (Pirmoradi et al., 2018; Smirnova et al., 2023; Liu et al., 2017). The secretome of MSCs with senescence-associated secretory phenotype (SASP) generates a proinflammatory microenvironment affecting surrounding cells (Siraj et al., 2023). ADSCs are more resistance to senescence shifts and comparative studies show long-term cultures have higher proliferation rates and decreased oxidative stress (Wagner et al., 2005; Wagner et al., 2008; Gruber et al., 2012). Additionally, hollow-fiber bioreactor systems retain differentiation capacity, surface phenotype, genetic stability, and show no evidence of increased aging after large-scale culture (Vymetalova et al., 2020). Our commercial-scale ADSC bioreactor process monitors glucose consumption, cell proliferation, and cell cycle to ensure that the cells are not senescent, thus safeguarding against SASP vesicle formation which may compromise the efficacy of the product.

Briefly, EVs are isolated from adipose-derived stem cells (ASCs) using a hollow-fiber cartridge containing bioreactor (Ludlow and Bueher, 2022). The ASCs used are isolated from human adipose tissue and expanded in culture using established protocols (Buehrer and Cheatham, 2013). To prepare the bioreactor, ASCs from multiple donors with similar demographics were seeded onto the cartridge. After seeding, the separate cell cartridge was filled with serum-free medium for ASC maintenance and EV production, and growth medium was continually circulated through the bioreactor with medium exchanges every 3–5 days. Bioreactor cultures can be maintained in this manner for up to 3 months while the ASCs retain their ISCT-mandated MSC characteristics.

EVs were collected every 3–5 days after the bioreactor culture was established. The conditioned serum-free medium was removed from the cell cartridge and subjected to two centrifugation steps as follows: The supernatant was aspirated, and the pelleted EVs containing exosomes were suspended in DPBS and stored at −80 °C for further analysis.

### 2.2 EV characterization methods

Particle size and concentration was determined using ZetaView NTA (Particle Metrix) instrumentation. Concentrated EV preps were diluted in particle free water to a range of 5 × 107 particles per milliliter and analyzed using defined acquisition settings. Size distribution was determined and mean, median, and mode diameters were used to characterize EVs.

Transmission electron microscopy was performed as follows: Size 300 mesh copper EM grids were glow-discharge formvar/carbon film coated for 45 s. Once completed, 5 µL of EV suspension (previously fixed with 30 µL of 2% PFA) was added to the grid and incubate for 1 min. Following removal of the excess EV solution, one drop of filtered 1% uranyl acetate was added and the excess removed before rinsing the grid on a drop of water 3 times. The grids were allowed to dry for 10 min at room temperature followed by imaging on Tecnai 12 electron microscope at 120 kV.

EV surface markers were analyzed using a Miltenyi MACSQuant Analyzer 10 for sample acquisition and MACSQuantify Software for data analysis. The MACSPlex Exosome Kit (Miltenyi Biotec) was used to evaluate each sample for the expression of 37 surface markers according to manufacturer’s protocol. Protein concentration was determined for each sample using BCA protein quantification kit (Thermo). 20 μg of prepared exosomes was gently rotated at 4 °C overnight with capture beads. The beads were washed and then incubated for 1 h with detection bead mixture consisting of CD9, CD63 and CD81 exosome markers. The flow cytometer was calibrated and background settings were adjusted to unlabeled beads and gating strategies were used to identify bead populations for each analyte. Batch analysis quantified median intensities for each bead population and analyte surface expression was calculated for each sample.

### 2.3 *In vitro* inflammatory cytokine assay

To measure EV effects on inflammation, *in vitro* gene expression for the inflammatory cytokines IL-6, IL-8 and MCP-1 was performed via RT-qPCR. Primary human fibroblasts (PHFs) were seeded at a density of 390,000 cells per well in growth medium (DMEM-LG, 10% FBS and antibiotics) using 6-well plates. The cells were incubated overnight to allow the PHFs to attach. To induce inflammatory cytokine production, growth medium was removed and replaced with Test Medium (DMEM-HG, ascorbic acid and antibiotics) containing 1 × 107/mL heat killed P. gingivalis (HKPG, InvivoGen) with and without 6.7 μg/mL ASC EVs. After overnight incubation, the cells were washed and RNA extracted using RNeasy Mini Kit (Qiagen). cDNA was prepared using 1 µg of purified RNA in a 20 µL reaction and High-Capacity cDNA Reverse Transcription Kit (Life Technologies) and qPCR performed with 10 ng cDNA per reaction using iTaq Universal Probes Master Mix (BioRad) and a QuantStudio 12K Flex machine (Life Technologies).

### 2.4 *In vitro* migration assay

Radius™ Cell Migration Assay plates were used for this assay. Briefly, plates were prepared and human primary skin epithelial cells were seeded (100,000 cells/well) into a 24-well Radius plate in 10% Serum containing Media (1 mL) as per manufacturer’s instructions. Following a 4h incubation at 37 °C in a 5% CO_2_ –containing atmosphere to allow cells to attach, wells were washed and the hydrogel dissolved from the wells. The wells were washed again, and either Serum Free, 1% Serum containing media, 10% serum containing media, or 1% serum containing media supplemented with 50 µL of indicated number of EVs. Photographs of each well were taken at T = 48 following addition of EVs.

### 2.5 *In vitro* cell proliferation assay

EVs were also added to low density *in vitro* PHF and primary human keratinocytes (PHKs) cultures (3,000 cells/well) in 96-well culture plates using serum-free medium and incubated for 3 days. To compare the proliferative effects of EVs, cells were treated with other inducers, including 10% FBS, PDGF-BB, KGF, TGF-β1, or IGF-1. After 3 days, the cells were treated with Cell Titer Blue reagent (Promega) for 2 h to assess proliferation by fluorescence signal (excitation: 560 nm, emission: 590 nm).

### 2.6 *In vitro* collagen production assay

Dermal fibroblasts were seeded at 10,000 cells per well and allowed to attach overnight. The next day cells were treated with EV and controls in assay medium +/− ascorbic acid. Cells were treated for 48 h in replicates of 3. After 48 h, conditioned media was collected and frozen at −80 °C until ready to assay. Procollagen-1 levels were determined by ELISA (Takara, cat# MK101) following the manufacturer’s instructions.

### 2.7 Excisional wound model

All animal husbandry is done in accordance to The Guide for the Care and Use of Laboratory Animals published by the Institute of Laboratory Animal Resources Commission on Life Science National Research Council, NATIONAL ACADEMY PRESS, Eighth Edition, and applicable USDA regulations without exception. Animals were housed on site (Synchrony Laboratories, Durham, NC, Study # 169-03-17) for a minimum of 5 days prior to study assignment. Animals were handled by staff daily to acclimate to human contact and reduce stress. Appropriate SOPs and protocols were followed for housing conditions, health status review, diet, pre-op instructions, and post-operative care. Marking and identification of animals was achieved by two non-invasive methods. 1. A black sharpie marker was used to inscribe a number on the fur. 2. Each animal was housed individually with identification number affixed to the cage. Both circular and linear skin injury models were developed and investigated. For circular wounds, 4x 2 cm diameter full thickness (entire skin layer) wounds were cut on the back of each animal. Sterile saline (phosphate buffered, PBS) was added to each wound and the injury covered with a transparent sterile, gas-permeable bandage or surgical tape. Each wound was measured and photographed daily for up to 30 days post-surgery. For linear wounds, 4x 2 cm linear full thickness cuts were made in the back skin. Sterile saline (phosphate buffered, PBS) was added to each wound and the injury covered with a transparent sterile, gas-permeable bandage or surgical tape. Each wound was measured and photographed daily for up to 30 days post-surgery. Baseline wound healing rates were established using measurements derived from these preliminary studies. Circular or linear wound healing models were generated on back skin of rodents as described above. Of the 4 wounds per rat, one was treated with PBS as a vehicle control. The other 3 were treated with an exosome preparation derived from adipocyte conditioned media. The treatments were randomized across subjects to avoid positional bias and assessment was blinded to avoid observer bias. Matched conditions on the same animal improved statistical power by allowing paired analysis. Up to 4 experimental groups (with different exosome preparations) were tested, 6 rodents per group. All rodents were evaluated for wound healing response as described above. At the end of the study period, rodents were euthanized and the wound site dissected out for evaluation by molecular and histological techniques.

### 2.8 Topical formulation (test article)

eXo^3^ exosomes are registered under International Nomenclature of Cosmetic Ingredients (INCI) name: Human Adipose Stromal Cell Exosome. eXo^3^ exosomes personal care formulations are registered in compliance with FDA under the Modernization of Cosmetics Regulation Act of 2022. The product described is not a drug product and was initially used solely to improve the condition and appearance of the skin. For all cell-based assays and animal studies, eXo^3^ exosomes were diluted in PBS and PBS alone was used as a vehicle control. For the patient case study, eXo^3^ Exosomes were blended with a base formulation consisting of 1.0% hyaluronic acid-based gel formulation developed by Exotropin, LLC and used as an Over The Counter (OTC) personal care product.

### 2.9 Diabetic wound healing in patient volunteer- a single case study

The presented case involves a 55-year-old male patient with a 30-year diagnosis of diabetes mellitus type 2 with stable A1C and other investigations within normal limits, presenting with multiple Grade 1 non-healing ulcers over the foot that had been previously treated for over 6 months by a foot surgeon. The surgeon had not been successful in healing these wounds using conventional therapy including debridement, off-loading with a soft cast, infection management and weekly dressing changes. Over the prior 6-month treatment period to promote healing, various dressing types were tried including alginate, silver, hydrocolloid and honey impregnated dressings. Despite active treatment, 3 large ulcers remained on the foot and one on the toe. Due to the patient’s history of chronic non-healing foot ulcers, an alternate solution was sought to accelerate healing.

Patient volunteer was treated once weekly with topical application of eXo^3^ exosome test article. Ulcers were thoroughly cleaned but not debrided. Exosome gel was applied topically directly to the wound bed, sufficiently to cover ulcers followed by Tegaderm to cover the gel as well as a secondary dressing to cushion and protect the treatment areas. The wounds were monitored regularly for signs of infection or complications but treatment and dressing were only changed and reapplied weekly. Foot wounds were photographed by care provider and ImageJ was used to detect open wound and quantify pixel area.

### 2.10 Statistical analysis

Quantitative data were expressed as mean ± standard deviation (SD) based on at least three independent assays in duplicates or triplicates. Students unpaired t-tests were performed using GraphPad Prism (GraphPad Company) for all *in vitro* assays. ANOVA was used to determine significance in the described animal wound healing study as well as in the human case study. Graphs are presented mean ± SD or mean ± s.e.m. All p-values below 0.05 (*), 0.01 (**), and 0.001 (***) were considered statistically significant.

## 3 Results

### 3.1 Extracellular vesicles characterization

**FIGURE 1:**
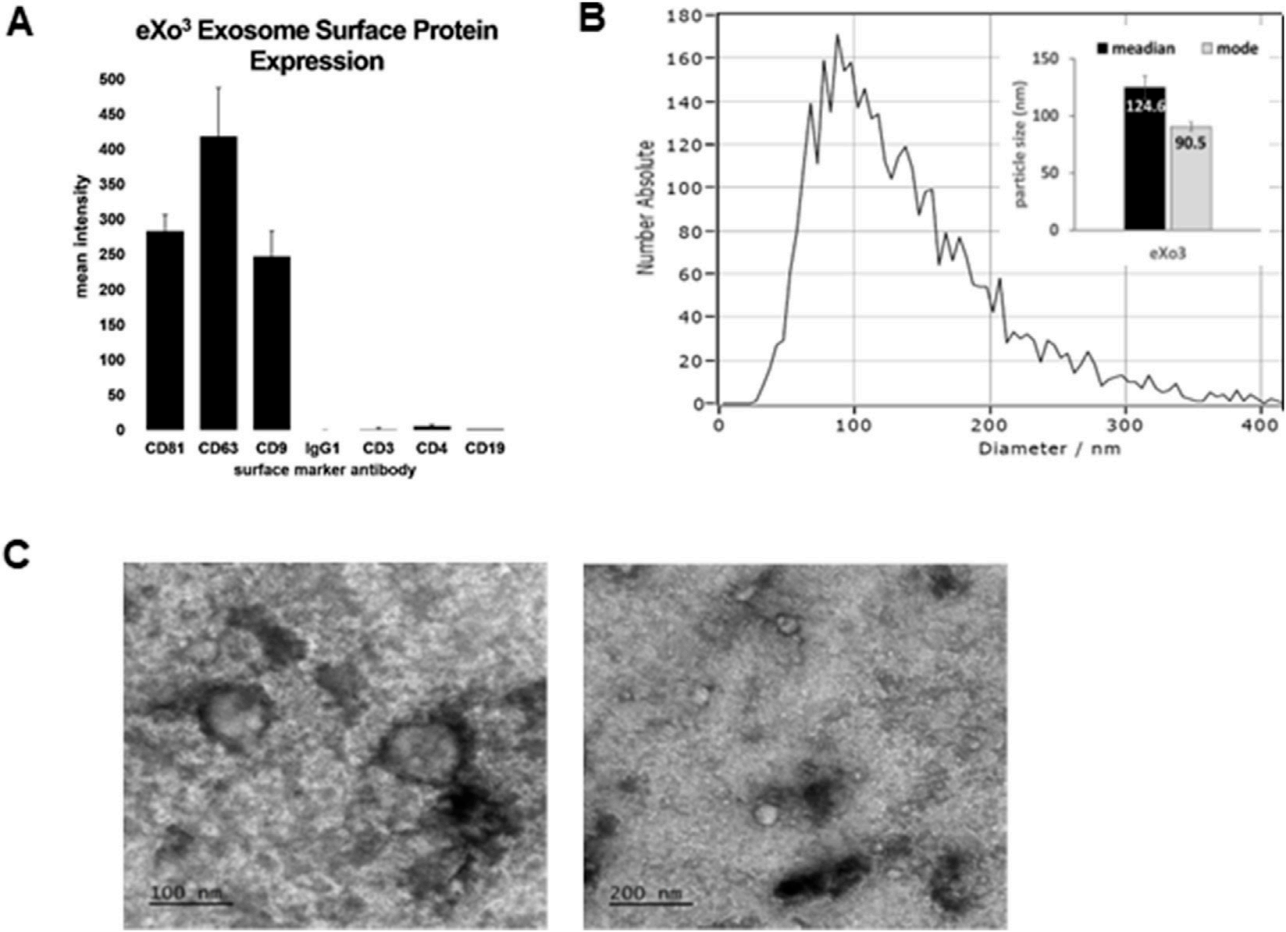
Manufactured EVs isolated from cultured ASCs were characterized using MISEV guidelines for EV classification. **(A)** Shows the expression of classical EV surface markers CD9, CD63, and CD81 detected in isolated eXo^3^ EVs. **(B)** Shows EV size distribution using ZetaView NTA. **(C)** Shows TEM images depicting bi-membrane vesicles sized at 80–140 nm in diameter.

The manufacturing process using ASC-derived EVs was adapted from previously published protocols (Basu and Ludlow, 2016). To ensure lot consistency, MISEV guidelines for EV characterization were followed using multiple methods for identification (Zhang et al., 2024; Welsh et al., 2024). Each lot was screened using flow cytometry to identify the presence of exosomes surface markers (Figure 1A). Purified particles were stained with antibodies targeting tetraspanin proteins CD9, CD63, and CD81. Positive identification was confirmed while negative expression of lymphocytic markers CD3, CD4, and CD19 illustrated preparation purity (Figure 1B). Particle size was determined using ZetaView NTA analysis showing a distribution range from 65 to 200 nm, consistent with published exosome biology (Gurunathan et al., 2021; Zhang et al., 2020). TEM was used to validate vesicle size and morphology, confirming isolation and purification of EVs (Figure 1C).

### 3.2 eXo^3^ exosomes stimulate collagen production and reduce inflammation

**FIGURE 2:**
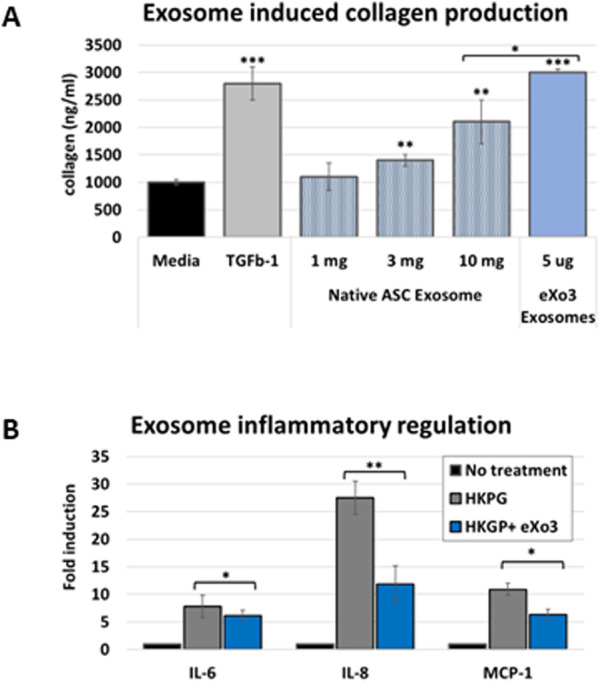
**(A)** Human dermal fibroblasts are treated with native or modified ASC-derived EVs. Media was harvested and analyzed for collagen accumulation. eXo^3^ EVs show significant stimulation of collagen production that is comparable to TGFb-1 treatment. **(B)** Dermal fibroblasts are stimulated with HKGB to activate an immune response. Co-incubated eXo^3^ EV samples show a restrains cytokine release detected by ELISA targeting IL-6, IL-8, and MCP-1. p-values below 0.05 (*), 0.01 (**), and 0.001 (***).

Chronic wound tissue is associated with degradation of the extracellular matrix and collagen fiber degeneration. The first stages of wound healing require collagen deposition to support extra cellular matrix formation which facilitates cell migration and tissue regeneration. We evaluated whether ASC-derived EVs could modulate collagen production using primary human fibroblasts (PHFs). EVs isolated from different controlled bioreactor microenvironments were applied to PHF cultures to stimulate collagen production. Serum-free conditioned medium was used for baseline comparison and TGFβ-1 was used as positive control. Native ASC EVs showed a dose dependent stimulation of collagen that did not reach levels of TGFβ-1 stimulation (Figure 2A). Interstingly, “tuned exosomes”, eXo^3^ exosome treatment was significantly higher than native EVs and matched a response similar to TGFβ-1. Figure 2B revealed reduced inflammatory cytokine production in response to exosome treatment.

### 3.3 eXo^3^ exosomes stimulate cell migration

**FIGURE 3:**
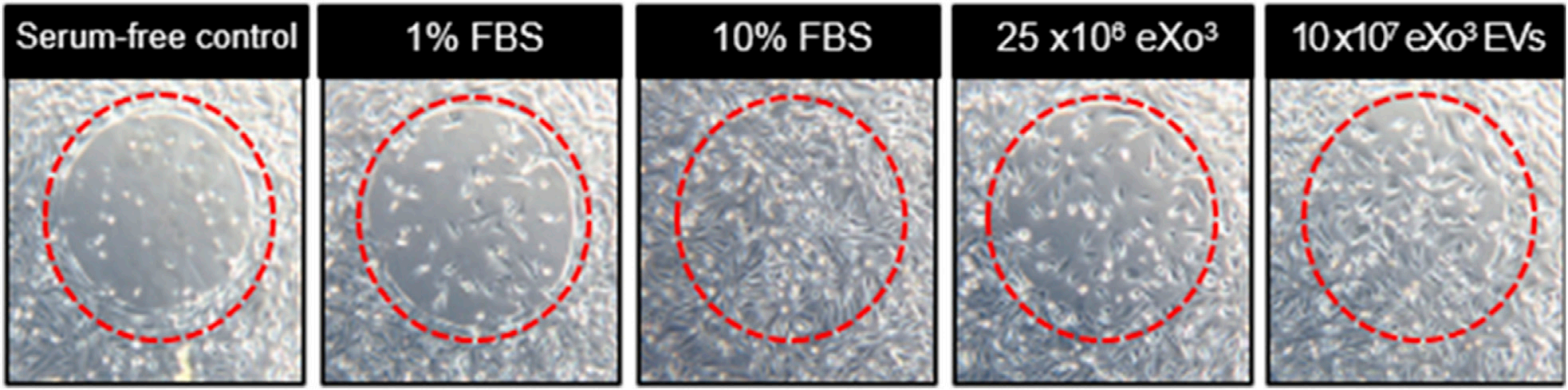
From left to right, serum free media serves as the negative control. 1% FBS media is the vehicle control. 10% FBS media is the positive control. Vehicle supplemented with EVs are shown in the last 2 panels.

When cells are seeded in the well of the Radius plate, they will adhere everywhere except in the center of the well where there is a biocompatible hydrogel spot of a defined and constant area. Once cells form a monolayer, the assay is initiated by gently dissolving the gel with a removal solution, leaving a gap across which cell migration can occur. As shown in Figure 3, at T = 48 h, serum free media (negative control) shows minimal migration. As expected, 1% FBS media shows slightly more migration while10% FBS media shows full closure of the gap. Addition of 25 × 10E6 EVs to the 1% FBS media reveals slightly better gap closure compared to the 1% FBS media alone, while 10 × 10E7 EVs elicits complete gap closure when added to the 1% FBS media.

### 3.4 eXo^3^ exosomes improve recovery in In vitro rodent wound healing study

**FIGURE 4:**
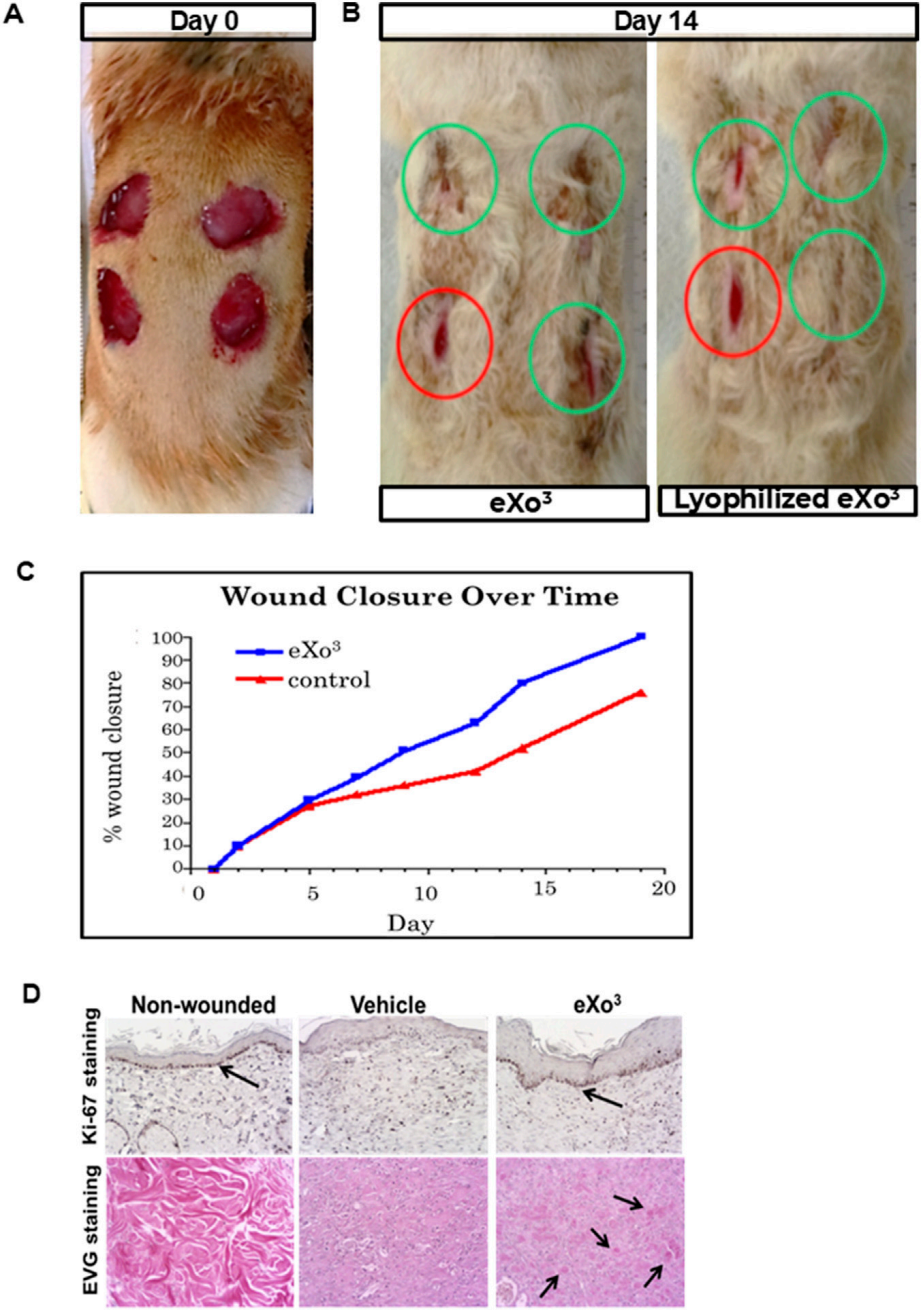
Rat wound healing model. **(A)** Four excisional wounds shown on animal at Day 0; **(B)** Same animal at Day 14 of treatement with either vehicle, eXo^3^ exosomes, or lyophilized eXo^3^ exosomes. Red circles-Vehicle; Green circles-eXo^3^ treatment. **(C)** Wound diameter was measured daily and percent closure data is graphed over 20 days. eXo^3^ treatment shows enhanced wound closure. ANOVA p < 0.05. **(D)** Histological sections of non-wounded, vehicle-treated, and eXo^3^-treated tissue, stained with Ki-67 and EVG show increased Ki-67 and collagen staining in eXo^3^-treated wounds. Differences in cellular activity and tissue structure are shown with arrows.

We used an excisional wound model in which 2 cm diameter wounds were created in the dorsal skin of Sprague-Dawley rats (Figure 4A) and were treated topically with a single dose of vehicle (300 µL) or HS-tuned ASC EVs (25 × 106 particles). The wounds were measured every 2–3 days to monitor the healing process. As shown in Figure 4B, the wound closure rate was increased by HS-tuned EV treatment (green) after 14 days compared to the vehicle control (red). Figure 4B demonstrates that HS-tuned EVs subjected to lyophilization (green) and room temperature storage for 7 days prior to reconstitution maintain their capacity to improve wound healing *in vivo*. Figure 4C is a graphic representation of the closure rates over time. Note that lyophilized EVs, when reconstituted with sterile water, resulted in a wound closure rate comparable to that of freshly-isolated EVs. The wound areas were excised after 19 days and analyzed by immunohistochemistry. EV-treated wound tissue demonstrated darker staining for Ki-67 (Figure 4D) than vehicle, suggesting that there is more proliferation in EV-treated wounds.

### 3.5 Human volunteer single case study: topical application of eXo^3^ exosomes promotes the appearance of ulcer healing

**FIGURE 5:**
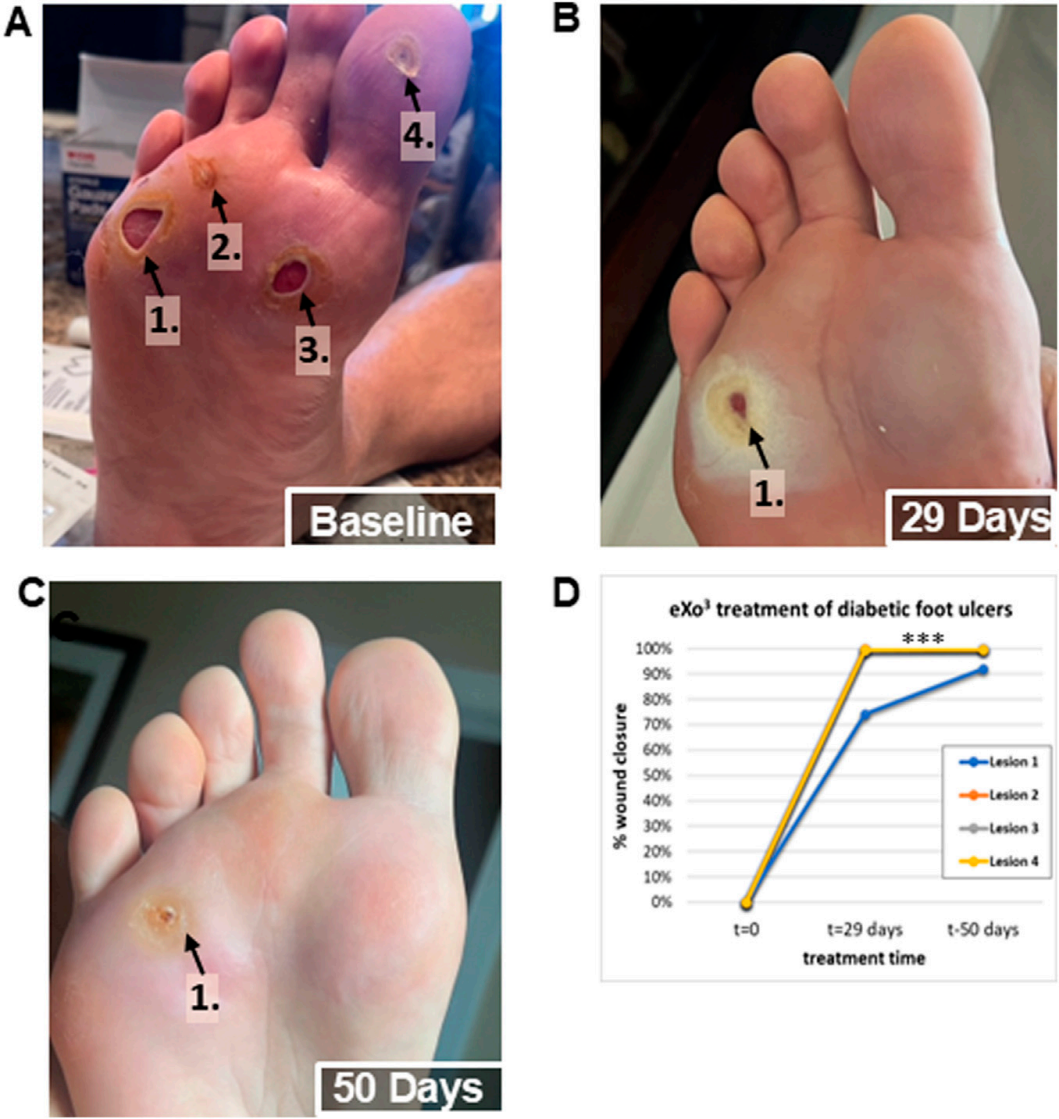
Human volunteer single case study observations after topical application of eXo^3^ exosomes to chronic diabetic ulcers. **(A)** Baseline: Non-healing diabetic ulcers after 6 months of treatment attempts with other therapeutic agents. **(B)** 29 Days: After 4 once weekly treatments with topical eXo^3^ exosomes formulated in a hyaluronic acid gel. **(C)** 50 Days: After 7 once weekly treatments with topical eXo^3^ exosomes formulated in a hyaluronic acid gel. **(D)** Digital image analysis of wound closure area over time of treatment.

Given that eXo^3^ significantly enhanced wound healing in the rodent model (Figure 4), we formulated the active ingredient for human skin application. The topical eXo^3^ exosomes formulated in a hyaluronic acid gel was initially used solely to improve the condition and appearance of the skin. However, when applied to subjects suffering from diabetic ulcers which were previously refractive to currently accepted and practiced methods of treatment, we saw dramatic effects. Figure 5 documents a human volunteer single case study where eXo^3^ exosome gel was applied topically directly to the wound bed. Figure 5A shows baseline wound state. The four ulcers were sufficiently covered with eXo^3^ gel followed by Tegaderm to cover the gel as well as a secondary dressing to cushion and protect the treatment areas. The wounds were monitored regularly for signs of infection or complications but treatment and dressing were only changed and reapplied weekly. Wound healing was documented after each dressing change. After 4 weeks of treatment, three lesions were completely healed, while the largest lesion showed >75% recovery (Figure 5B). Treatment was continued for 3 more weeks and resulted in nearly 100% recovery (Figures 5C,D). Quantitative image analysis of identified lesions in images identified open wound area and calculated the lesion size. Each wound site was normalized to baseline area at time 0 and the percent closure was calculated through out the term of collection These reported findings were unexpected; however, they support the ability to “tune” pro-healing EVs that provide therapeutic benefit beyond traditional pharmacological treatments. While encouraging in this single human volunteer case study, we recognize that further investigation is warranted to strengthen these anecdotal results.

## 4 Discussion

Stem cell therapies represent a compelling means of tissue repair and have demonstrated wound healing and soft tissue regeneration in animal models. Stem cell therapeutic approaches have the advantage of being capable of potentially regenerating an assortment of tissues by virtue of their pluripotency. Such versatility is a much-needed option for various medical conditions. The downsides of stem cell therapy include their complexity in isolation, characterization, and patient delivery. The greatest concern continues to be the potential for uncontrolled cell growth, proliferation, and tumorigenesis. The use of embryonic stem cells for therapeutic applications also raises ethical concerns and continues to be a passionately debated topic. Additionally, the intuitive concept that therapeutic stem cells engraft and differentiate at sites of tissue damage is not well supported given the low numbers of cells retained over time at application sites *in vivo* (Lotfy et al., 2023). This suggests that their mechanisms of action occur through paracrine modalities such as secretion of bioactive vesicles, including exosomes. Hence, exploiting stem cell-derived exosomes as a biologic-derived therapy, rather than delivering transient stem cells to treat chronic wounds, is an enticing approach.

Secreted extracellular vesicles (EVs), such as exosomes, are packed with potent pro-repair proteins and RNA cargos that are both cell type-specific, as well as, differentially produced and secreted according to the cellular environment (Zhang et al., 2019; Kim et al., 2017; Qiu et al., 2020). Exosomes offer a novel approach to tissue engineering and regenerative medicine therapies having several advantages over the use of cells. Since exosomes are cell-free, there is a much lower risk of immune rejection allowing for their allogenic use in the clinic. Exosome regenerative and wound healing capabilities are also heightened by having an elevated concentration of growth factors compared to cells and manufacturing large-scale, GMP-grade product is accomplished at sustainably lower costs. While encouraging, it needs to be recognized that exosome therapy is still early in development, and regulatory considerations such as purity, batch-to-batch consistency, and potency need to be addressed before widespread adoption for clinical use. The data presented here supports the idea that manipulation of the cellular bioreactor environment improves the repair activity of stem cell-derived EVs *in vitro* and *in vivo*. Current investigation into the cell-signaling elicited by these EVs will facilitate further understanding of the mechanisms involved in wound healing and tissue repair. Towards this goal, we have partially analysed miRNA cargo contained in eXo^3^ and found several sequences implicated in wound healing. Additionally, current proteomic research has identified cell cycle regulatory proteins and stress response proteins such as HSP70 that may contribute to the observed pro-healing function of eXo^3^. Once our analysis is complete, we will report on the miRNA and protein cargos we believe to be important for this healing effect and potentially lead to additional tissue engineering and regenerative medicine applications.

The singe human volunteer case study reported herein shows promising therapeutic benefit and wound healing potential of eXo^3^ application. Diabetic foot ulcers remain a therapeutic area of high medical unmet need. The preclinical data presented, including the animal studies, combined with the morbidity and mortality associated with diabetic foot ulcers served to highlight the critical need to treat this patient. Given that 70% of diabetic foot ulcers remain unhealed at 20 weeks, the rapid closure of these wounds is significant. The rationale for why this patient was treated as well as the successful healing presented will hopefully serve as justification for larger clinical trials in this area of unmet need. Follow-up studies over time are required to adequately assess the effectiveness of eXo^3^ in its current product formulation. If eXo^3^ were to be administered in a manner other than topically, such as oral ingestion, subcutaneous or systemic injection, the product must be manufactured in a cGMP compliant manner following the guidelines put forth by the FDA for clinical trial products. While we are not planning to submit a clinical trial application at this time, there are currently 134 exosome-based studies investigating EV therapeutic use according to ClinicalTrials.gov (National Library of Medicine, 2025). With a growing number of studies focused on wound healing, our data presented and research efforts provide valuable translational evidence supporting the therapeutic benefits of exosomes (Hu et al., 2016; Qiu et al., 2020; Shabbir et al., 2015; Shi et al., 2021).

## 5 Conclusion

The innovation demonstrated here focuses on co-opting the cellular context-dependence of ASC loading and secretion of EVs for engineering a potentially novel pro-healing therapeutic. Here we also show that “tuned” EVs from adipose derived stem cells have the ability to facilitate accelerated and improved skin wound healing upon application in a topical formulation. The bioreactor-based production approach enables large quantities of exosomes to be isolated from relatively small volumes of conditioned culture media, when compared to culturing in traditional culture plastic ware generating multiple liters of media. The bioreactor is also considered a “closed system”, thus addressing at least one recommendation by regulatory agencies for products derived from cell culture. This approach reduces the cost of goods for large scale applications, such as OTC personal care products and potential FDA-allowed medicinal products.

## Data Availability

The raw data supporting the conclusions of this article will be made available by the authors, without undue reservation.
